# Human Papillomavirus Viral Load as Triage Biomarker for High‐Grade Cervical Lesions and Invasive Cervical Carcinoma: A Cross‐Sectional Study

**DOI:** 10.1002/hsr2.71524

**Published:** 2025-11-17

**Authors:** Mariem Salma Abdoudaim, Laurent Bélec, Mohamed Lemine Cheikh Brahim Ahmed, Nacer Dine Mohamed Baba, Ralph‐Sydney Mboumba Bouassa, Mohamed Vall Mohamed Abdellahi

**Affiliations:** ^1^ Unité d'Epidémiologie Moléculaire et Diversité des Microorganismes, Faculté des Sciences et Techniques de l'Université de Nouakchott Nouakchott Mauritania; ^2^ Laboratoire de virologie, Hôpital Européen Georges Pompidou, Assistance Publique‐Hôpitaux de Paris Paris France; ^3^ Faculté de Médecine Paris Descartes, Université Paris Cité Paris France; ^4^ Centre Hospitalier National Nouakchott Mauritania; ^5^ Institut du Savoir Montfort, Montfort Hospital, Department of Family Medicine, Faculty of Medicine University of Ottawa Ottawa Canada; ^6^ Ecole Doctorale Régionale (EDR) d'Afrique Centrale en Infectiologie Tropicale Franceville Gabon

**Keywords:** cervical cancer, cervical pathology, HPV, HPV viral load, mauritania, multiplex real‐time PCR

## Abstract

**Background and Aims:**

We herein evaluated whether intra‐tissue HPV viral load may constitute a triage biomarker to differentiate between high‐grade precancerous cervical lesions from intra cervical cancer (ICC).

**Methods:**

50 biopsy samples prospectively obtained from women living in Mauritania suffering from high‐grade cervical intraepithelial neoplasia (CIN2/3), adenocarcinoma (ADC) or squamous cell carcinoma (SCC) were analysed for HPV genotyping and quantitation carried out using Bioperfectus Multiplex Real Time Human Papillomavirus Genotyping Real Time PCR assay.

**Results:**

HPV‐positive results were detected in 47 biopsies (12 CIN2/3 and 35 ICC, including 4 ADC and 31 SCC). The cumulative HPV viral loads of any HPV and high risk‐HPV (HR‐HPV) in ICC were significantly higher than those in CIN2/3 (*p* < 0.002 for any HPV;  0.02 for HR‐HPV). The cumulative viral loads of any HPV and HR‐HPV possessed a good discriminatory ability to differentiate between CIN2/3 and ICC, with optimal cutoffs ranging from 4.38 (any HPV) to 4.85 (HR‐HPV) copies per 10,000 cells.

**Conclusion:**

Our observations show that cumulative HPV viral load in cervical tissue may constitute a relevant biomarker associated with the severity of HPV‐related cervical lesions. HPV viral load in cervical tissue could be used as a triage tool for aggressive ICC in advanced cervical lesions.

## 1 Introduction

According to the World Health Organization (WHO), about 660,000 new cases of cervical cancer and 350,000 deaths were reported in 2022, making cervical cancer the ninth deadly female cancer worldwide [1]. With almost 150,000 new cases and 100,000 deaths per year, cervical cancer associated with high risk‐human papillomavirus (HR‐HPV) constitutes a major public health problem in sub‐Saharan Africa, ranking the second most common cause of female cancer incidence and mortality in African women [2, 3].

HPV DNA detection by molecular biology is now recommended as the primary screening method in cervical cancer prevention, as indicated by the World Health Organization (WHO) guidelines [4], with the advantage of higher clinical sensitivity and negative predictive value, as compared to pathology, but with a lower clinical specificity [5]. A variety of molecular biomarkers have been recognized which predict the outcome of HR‐HPV infection by roughly identifying specific stages in the natural history of HPV infection and cervical cancer progression to identify among persistently infected women those at risk to develop cervical cancer [6]. The HPV viral load of oncogenic HPV, defined as the quantity of HPV genomes present in a cervical sample or cervical tissue, has become an attractive potential routine biomarker [7, 8]. Since the standardization of viral load determination by multiplex PCR [8, 9, 10], HPV viral load may be used as an easy surrogate biomarker of potentially great value in improving treatment strategies and thereby possibly clinical outcome. Indeed, multiplex PCR offers significant advantages for the diagnosis, genotyping, and quantitation of HPV due to its ability to simultaneously target multiple HPV types or different regions of the HPV genome in a single reaction. Thus, multiplex PCR can detect and identify the presence of multiple HPV types, including both high‐risk and low‐risk variants, in a single assay. This significantly reduces the time, cost, and sample volume required compared to running individual PCRs for each type. It also allows for the screening of a wide range of HPV genotypes, increasing the chances of detecting any infection present, even if it involves multiple types. Furthermore, the multiplex PCR assay includes internal control to ensure the reliability of the test by confirming successful DNA extraction and amplification, reducing the possibility of false negatives. Finally, by including primers for a ubiquitous cellular reference gene [e.g., β‐globin or human DNA topoisomerase 3 (TOP3)], multiplex PCR can provide a semi‐quantitative measure of the HPV viral load relative to the amount of host DNA, that can be important for monitoring persistent infections and assessing the risk of disease progression. Thus, HPV viral load has been proposed as a diagnostic marker of ongoing cervical precancerous and cancerous lesions, with an overall viral load increase with the severity of cervical tissue injuries [8, 11]. Furthermore, in African resource‐constrained countries, frequently lacking access to pathological facilities [12], the application of appropriate viral load cutoff values could be used as an effective triage tool to improve the specificity of detection in cervical cancer screening and to care HR‐HPV‐positive women.

Cervical cancer related to HR‐HPV is the second female cancer in Mauritania, a country in Northwest Sahelian Africa with less than 5 million inhabitants. According to the WHO, with an incidence rate of 14.3% (468 new cases) and a mortality rate of 13.5% (302 deaths) in 2022, cervical cancer ranks as both the 2nd most frequent and most deadly female cancer in Mauritania [13]. We recently evaluated by molecular biology the HPV genotypes distribution in Mauritanian women suffering from high‐grade cervical intraepithelial neoplasia (CIN2/3) or invasive cervical cancers (ICC), including adenocarcinoma (ADC) and squamous cell carcinoma (SCC), with the perspective of prophylactic vaccination against HPV [13]. We were able to complete and extent these epidemiological observations by measuring HPV viral load in biopsy specimens. The aim of this study was to evaluate in an African context whether intra‐tissue HPV viral load may constitute a triage biomarker to differentiate between high‐grade precancerous (CIN2/3) lesions from ICC.

## 2 Materials and Methods

### 2.1 Study Design and Participants, Collection and Processing of Biopsy Samples

Between 2022 and 2023, adult women referred to the *Centre Hospitalier National*, Nouakchott, Mauritania, for suspected high‐grade lesion or cervical cancer were prospectively recruited after informed consent. A total of 50 women diagnosed histologically with either CIN2/3, ADC or SCC, were included in the study. Socio‐demographic characteristics from included patients, including age, clinical data, previous HPV screening and vaccination, were previously reported [13]. In brief, the mean age of the study population was 56.7 years (range, 35–73 years). All women lived in the urban setting of Nouakchott. Most women (40.0%) were aged 60–69 years, and the second most frequent age group (26.0%) was women aged 50–59 years. Most of them were engaged in life couple with a male partner (80.0%), with a low education level (78%), half of them (48%) had never been to school. All women were multiparous, with a mean number of 4 children (range, 2–6). Most of them were postmenopausal (84.0%). Only a minority (18.0%) of study women had previously received HPV cervical screening, mainly those diagnosed with CIN2/3 (26.4%). Note that none of the study's women had ever received prophylactic HPV vaccination and none of them were infected with HIV.

Cervical biopsies were performed before treatment for CIN2/3 and ICC through colposcopy examination for histological analysis at the pathology laboratory of the *Centre Hospitalier National*. After being fixed overnight in 10% formalin, the biopsies were embedded in paraffin. The formalin‐fixed, paraffin‐embedded (FFPE) blocks were further processed using standard histopathological methods and evaluated independently by two certificated pathologists, the first reading being validated by an independent counter‐reading by a second pathologist.

Sections of 5–20 µm thick were cut from each FFPE block, placed on a slide for molecular investigations, and sent to the virology laboratory of the *hôpital europèen Georges‐Pompidou*, Paris, France.

### 2.2 HPV Viral Load Quantification

Sections of FFPE biopsy samples were deparaffinized overnight, and DNA was further extracted using QIAamp® DNA Mini Kit (Qiagen, Hilden, Germany) and stored at −20°C until analysis, as previously described [13].

HPV detection and genotyping was carried out using the fluorescence‐based Bioperfectus Multiplex Real Time (BMRT) Human Papillomavirus Genotyping Real Time PCR Kit (Jiangsu Bioperfectus Technologies Co. Ltd., Taizhou, Jiangsu Province, China; catalogue number: JC80301NW‐48T), according to manufacturer's instructions. The BMRT HPV assay amplifies short L1 gene sequences [9], as well as an internal control with the housekeeping single‐copy gene encoding human DNA topoisomerase III [14] in reaction tube H (FAM™ channel) to identify possible PCR inhibition and to confirm the reliability of the reagents in this kit as well as the viral loads simultaneously. According to the HPV classification nomenclature provided by the International Agency for Research on Cancer [15], the BMRT HPV Genotyping Real Time PCR assay allows to distinguish qualitatively each of the 21 most prevalent HPV genotypes, including 13 h‐HPV (HPV‐16, −18, −31, −33, −35, −39, −45, −51, −52, −56, −58, −59 and −68), 5 possibly oncogenic‐HPV (PO‐HPV) (HPV‐26, −53, −66, −73 and −82) and 3 low risk‐HPV (LR‐HPV) (HPV‐6, −11 and −81). Briefly, eight reactions per sample were performed simultaneously. Among them, the reactions A, B, C, D, E, F and G were prepared to detect and differentiate in FAM™/VIC® (HEX)/ROX™ fluorescent channels, HPV‐16/−18/−31, HPV‐59/−66/−53, HPV‐33/−58/−45, HPV‐56/−52/−35, HPV‐68/−51/−39, HPV‐73/−26/−82 and HPV‐6/−11/−81, respectively.

To validate the PCR reaction, the cycle threshold (Ct) values of positive controls had to be less than or equal to the cut‐off value of 30.0 in FAM™, VIC® (HEX) and ROX™, and the blank controls had to be undetectable. The optical unit of the real‐time PCR system measured the emitted fluorescence. For each of the 21 detected HPV genotypes, the qualitative reference values of positive cut‐off were assessed by the manufacturer using receiver operating characteristic (ROC) curves based on clinical trial results. Specimens with Ct values less than or equal to the cut‐off value of one given HPV type were considered as positive for this HPV genotype. The cut‐off values for Ct were those provided by the manufacturer in the instructions for use, ranging for positivity from 34.6 to 36.9 in FAM™, from 35.5 to 37.0 in VIC® (HEX) and from 34.5 to 35.6 in ROX™. Conversely, specimens with Ct values above the cut‐off value of one given HPV type were considered as negative.

The BMRT HPV Genotyping Real Time PCR assay allows to determine the normalized HPV viral load of the 21 detected HPV, expressed as log (normalized viral copies per 10,000 human cells), by integrating the Ct values of the emitted fluorescence of L1 gene of each HPV and the Ct of the sample TOP3 gene, using the Bioperfectus HPV Analyser Software v1.0 (Jiangsu Bioperfectus Technologies Co. Ltd.), as described [9].

### 2.3 Statical Analysis

Data were entered into an Excel database. Quantitative data were described by mean ± standard deviation. HPV viral load in biopsy specimens was compared by Kruskal–Walli's rank sum test for quantitative variables. ROC curves were constructed and used to assess the performances of the HPV viral load as assessed by BMRT HPV Genotyping Real Time PCR assay for predicting ICC among high‐grade precancerous and cancerous lesions using the type‐specific log‐transformed HPV viral loads at different positivity thresholds, and to identify the optimal cutoff value of the type‐specific HPV viral loads for predicting ICC. The area under the ROC curve (AUC) was calculated according to the method of DeLong et al. [16] as a measure of discriminative ability [17] and was expressed as mean ± standard error (with binomial exact 95% confidence interval). Higher AUC values were considered to demonstrate better discriminatory abilities as follows: excellent discrimination, AUC of ≥ 0.90; good discrimination, 0.80 ≤ AUC < 0.90; fair discrimination, 0.70 ≤ AUC < 0.80; poor discrimination, AUC of 0.60 ≤ AUC < 0.70; and fail discrimination, 0.50 ≤ AUC < 0.60 [17]. The hypothesis that the ratio of cases in the positive (ICC) and negative (CIN2/3) groups reflects the prevalence of the disease was chosen for the calculation of positive predictive values (PPV) and negative predictive values (NPV). For each AUC, the probability that a randomly selected individual from the positive (ICC) group has a HPV viral load indicating greater suspicion than that for a randomly chosen individual from the negative (CIN2/3) group was calculated, with corresponding P‐value [17, 18]. The optimal cutoffs of HPV viral load for identifying ICC were calculated for each ROC curve according to the disease prevalence in study specimens and the best clinical sensitivity and specificity to assess the best diagnosis accuracy. The sensitivity, specificity, positive likelihood ratio (PLR), negative likelihood ratio (NLR), PPV and NPV were determined for each optimal cutoffs of discriminating HPV viral load. The accuracy of HPV viral load to correctly diagnose ICC from high‐grade precancerous and cancerous lesions was estimated by Youden's J index (J = Sensitivity + Specificity − 1) [19], and by the overall accuracy considered the disease prevalence using the following formula: (Prevalence) (Sensitivity) + (1‐prévalence) (Specificity) [20]. In case of coinfections, HPV viral load used in the evaluation was the cumulative HPV loads in tissue specimen. A two‐tailed *P*‐value less than 0.05 was considered statistically significant. MedCalc® version 23.0.9 (MedCalc Sofware Ltd, Ostend, Belgium) was used for all statistical analyses.

### 2.4 Ethics Statement

This study was conducted in accordance with the ethical guidelines of the World Medical Association Declaration of Helsinki. Signed informed written consent was obtained by each participant after explaining the purpose of the cervical biopsy for histological and virological analyses. This study study is with a direct individual benefit, with the histological diagnosis of suspected cervical lesions and virological diagnosis of HPV detection and genotyping. The protocol was reviewed and approved by the Institutional Review Board and Ethics Committee of the Nouakchott University, Nouakchott, Mauritania (approval no. 004/CE/UN on 27 January 2022).

## 3 Results

### 3.1 Overall Prevalences of Hpv Genotypes and HPV Viral Load Distribution

HPV‐positive results were detected in 47 biopsies (94.0%), including 12 (85.7%) CIN2/3 and 35 (97.2%) ICC, with 4 ADC and 31 SCC, as reported previously [13].

The Table 1 depicts the mean cumulative HPV viral loads assessed by BMRT HPV Genotyping Real Time PCR assay according to the grade of cervical lesions for any HPV, HR‐HPV, HR‐HPV plus PO‐HPV, PO‐HPV plus LR‐HPV and vaccine HPV. In all comparisons, except for PO‐HPV plus LR‐HPV, the mean HPV viral loads were significantly higher in ICC than CIN2/3 (*p* <  0.002 for any HPV; < 0.02 for HR‐HPV; < 0.03 for HR‐HPV plus PO‐HPV; < 0.04 for vaccine HPV) as well as in women older than 50 years (< 0.01). The mean HPV viral loads for any HPV, HR‐HPV and vaccine HPV were significantly higher in ADC than CIN2/3 (*p* < 0.03, < 0.03 and < 0.04, respectively), as well as in women older than 50 years (< 0.02). The mean HPV viral loads for any HPV, HR‐HPV and Gardasil‐9® vaccine HPV were significantly higher in SCC than CIN2/3 (*p* < 0.005, < 0.003 and < 0.05, respectively), as well as in women older than 50 years (< 0.01). Gardasil‐9® vaccine HPV genotypes as well as HPV‐39 were more frequently detected in ICC, ADC, SCC than CIN2/3 (Table 2). Single any HPV genotype was more frequently detected in CIN2/3 than in tumour biopsy with only one HPV, and in ICC than CIN2/3 for HPV‐39, except in case of single HR‐HPV or PO‐HPV detection (Table 2).

**Table 1:** Comparisons of cumulative type‐specific viral loads of any HPV, HR‐HPV, HR‐HPV plus PO‐HPV, PO‐HPV plus LR‐HPV and vaccine HPV, in HPV‐infected women suffering from CIN2/3 and ICC.

	CIN2/3£	ICC£					
		ICC	*p*‐valueµ	ADC	*p*‐value$	SCC	*p*‐value£
**LR‐HPV,** **HR‐HPV** **and PO‐HPV** *	4.10 ± 0.70 (*n* = 12)	5.26 ± 1.16 (*n* = 35)	< 0.002	5.25 ± 0.14 (*n* = 4)	0.021	5.21 ± 1.20 (*n* = 31)	< 0.005
**HR‐HPV** **	4.05 ± 0.68 (*n* = 12)	5.20 ± 1.15 (*n* = 35)	< 0.02	5.25 ± 0.14 (*n* = 4)	< 0.02	5.15 ± 1.19 (*n* = 31)	< 0.003
**HR‐HPV** **(age** < **50 years)**	4.41 ± 0.40 (*n* = 5)	4.86 ± 1.04 (*n* = 6)	NS	4.27 (*n* = 1)	NS	4.98 ± 1.12 (*n* = 5)	NS
**HR‐HPV** **(age** > **50 years)**	3.80 ± 0.75 (*n* = 7)	5.21 ± 1.23 (*n* = 29)	< 0.005	5.47 ± 0.39 (*n* = 3)	< 0.02	5.18 ± 1.30 (*n* = 26)	< 0.008
**HR‐HPV plus** **PO‐HPV** ***	4.07 ± 0.70 (*n* = 12)	5.21 ± 1.21 (*n* = 35)	< 0.006	5.25 ± 0.82 (*n* = 4)	< 0.02	5.22 ± 1.27 (*n* = 31)	< 0.01
**PO‐HPV plus** **LR‐HPV** ***	3.59 ± 1.06 (*n* = 4)	4.53 ± 1.22 (*n* = 7)	NS	NA	NA	4.53 ± 1.22 (*n* = 7)	NS
**Vaccine HPV** ****	4.17 ± 0.63 (*n* = 11)	5.00 ± 1.25 (*n* = 33)	< 0.03	5.25 ± 0.14 (*n* = 4)	< 0.04	4.94 ± 1.16 (*n* = 29)	< 0.05
**Vaccine HPV** **(age** < **50 years)**	4.50 ± 0.39 (*n* = 4)	4.86 ± 1.04 (*n* = 6)	NS	4.27 (*n* = 1)	NA	4.98 ± 1.12 (*n* = 5)	NS
**Vaccine HPV** **(age** > **50 years)**	3.95 ± 0.69 (*n* = 7)	5.21 ± 1.27 (*n* = 27)	< 0.006	5.47 ± 0.39 (*n* = 3)	< 0.02	5.18 ± 1.35 (*n* = 24)	< 0.01

* Any HPV include the following 21 HPV genotypes (HPV‐6, ‐11, −16, −18, −26, −31, −33, −35, −39, −45, −51, −52, −53, −56, −58, −59, −66, −68, −73, −81, and −82).

** HR‐HPV include the following 13 h‐HPV genotypes (HPV‐16, −18, −31, −33, −35, −39, −45, −51, −52, −56, −58, −59, and − 68).

*** PO‐HPV include HPV‐26, −53, −66, −73 and −82; LR‐HPV include HPV‐6, −11 and −81.

**** Gardasil‐9® vaccine HPV include the following HPV genotypes (HPV‐6, −11, −16, −18, −31, −33, −45, −52, and −58).

^£^ Mean cumulative HPV viral load in log copies per 10,000 cells ±standard deviation.

^µ^ Comparison between CIN2/3 and any ICC by Kruskal–Walli's rank sum test.

^$^ Comparison between CIN2/3 and any ADC by Kruskal–Walli's rank sum test.

^£^ Comparison between CIN2/3 and any SCC by Kruskal–Walli's rank sum test.

Abbreviations: ADC, Adenocarcinoma; CIN, Cervical intraepithelial neoplasia; HR‐HPV, High risk‐HPV; ICC, Invasive cervical cancers; LR‐HPV, Low risk‐HPV; *n*, Number; NA, Not attributable; NS, Not significant; PO‐HPV, Possibly oncogenic‐HPV; SCC, Squamous cell carcinoma.

**Table 2:** Comparisons of cumulative type‐specific viral loads of single and multiple HPV genotypes in HPV‐infected women suffering from CIN2/3 and ICC.

	CIN2/3£	ICC£					
		ICC	*p*‐valueµ	ADC	*p*‐value$	SCC	*p*‐value£
**HPV‐45**	3.84 ± 0.53 (*n* = 5)	4.44 ± 1.47 (*n* = 15)	NS	5.92 (*n* = 1)	NA	4.33 ± 1.46 (*n* = 14)	NS
**HPV‐16**	4.47 ± 0.41 (*n* = 4)	4.77 ± 1.28 (*n* = 14)	NS	4.10 ± 1.13 (*n* = 3)	NS	4.65 ± 1.63 (*n* = 11)	NS
**HPV‐39**	3.46 ± 0.55 (*n* = 3)	5.66 ± 0.81 (*n* = 8)	< 0.02	5.03 (*n* = 1)	NA	5.75 ± 0.83 (*n* = 7)	< 0.02
**HPV‐52**	3.91 ± 0.88 (*n* = 2)	4.93 ± 1.83 (*n* = 9)	NS	3.18 (*n* = 1)	NA	5.15 ± 1.82 (*n* = 8)	NS
**HPV‐33**	4.37 (*n* = 1)	3.60 ± 1.31 (*n* = 6)	NA	2.36 (*n* = 1)	NA	3.85 ± 1.31 (*n* = 5)	NA
**HPV‐18**	4.56 (*n* = 1)	4.37 ± 0.55 (*n* = 6)	NA	5.08 (*n* = 1)	NA	4.14 ± 0.37 (*n* = 5)	NA
**HPV‐35**	NA (*n* = 0)	5.07 ± 0.58 (*n* = 2)	NA	NA (*n* = 0)	NA	5.07 ± 0.58 (*n* = 2)	NA
**HPV‐56**	NA (*n* = 0)	2.56 (*n* = 1)	NA	NA (*n* = 0)	NA	2.56 (*n* = 1)	NA
**HPV‐73**	5.41 ± 1.16 (*n* = 2)	4.67 ± 2.11 (*n* = 2)	NS	NA (*n* = 0)	NA	4.67 ± 2.11 (*n* = 2)	NS
**HPV‐53**	NA (*n* = 0)	4.14 (*n* = 1)	NA	NA (*n* = 0)	NA	4.14 (*n* = 1)	NA
**HPV‐81**	3.09 ± 1.06 (*n* = 2)	4.07 ± 1.14 (*n* = 3)	NS	NA	NA	4.07 ± 1.14 (*n* = 3)	**NS**
**Single** **HR‐HPV or** **PO‐HPV**	3.81 ± 0.59 (*n* = 7)	4.46 ± 0.67 (*n* = 16)	< 0.05	5.15 (*n* = 1)	NA	4.41 ± 0.61 (*n* = 15)	NS
**Double** **HR‐HPV or** **PO‐HPV (*n*** **=** **2)**	NA (*n* = 0)	5.69 ± 1.42 (*n* = 12)	NA	4.81 ± 0.76 (*n* = 2)	NA	5.89 ± 1.40 (*n* = 10)	NA
**Multiple** **HR‐HPV or** **PO‐HPV (*n*** ≥ **2)**	NA (*n* = 0)	5.82 ± 1.22 (*n* = 20)	NA	5.18 ± 0.83 (*n* = 3)	NA	5.94 ± 1.26 (*n* = 17)	NA
**HPV‐45 plus** **HPV‐16**	4.19 ± 0.59 (*n* = 8)	4.90 ± 1.32 (*n* = 22)	NS	5.11 ± 0.82 (*n* = 3)	NS	4.90 ± 1.32 (*n* = 19)	NS

^£^ Mean cumulative HPV viral load in log copies per 10,000 cells ±standard deviation.

^µ^ Comparison between CIN2/3 and any ICC by Kruskal–Walli's rank sum test.

^$^ Comparison between CIN2/3 and any ADC by Kruskal–Walli's rank sum test.

^£^ Comparison between CIN2/3 and any SCC by Kruskal–Walli's rank sum test.

Abbbreviations: ADC, Adenocarcinoma; CIN, Cervical intraepithelial neoplasia; HR‐HPV, High risk‐HPV; ICC, Invasive cervical cancers; n, Number; NA, Not attributable; NS, Not significant; PO‐HPV, Possibly oncogenic‐HPV; SCC, Squamous cell carcinoma.

Mean HPV viral load in SCC and ADC were otherwise similar (not shown). Finally, comparisons according to the detected HR‐HPV genotype, especially the two main study HR‐HPV (HPV‐45, HPV‐16) as well as HPV‐45 plus HPV‐16, could not reach statistical significance (Table 2).

### 3.2 Performance and Cutoff Values of HPV Viral Loads for Predicting ICC

**Figure 1:**
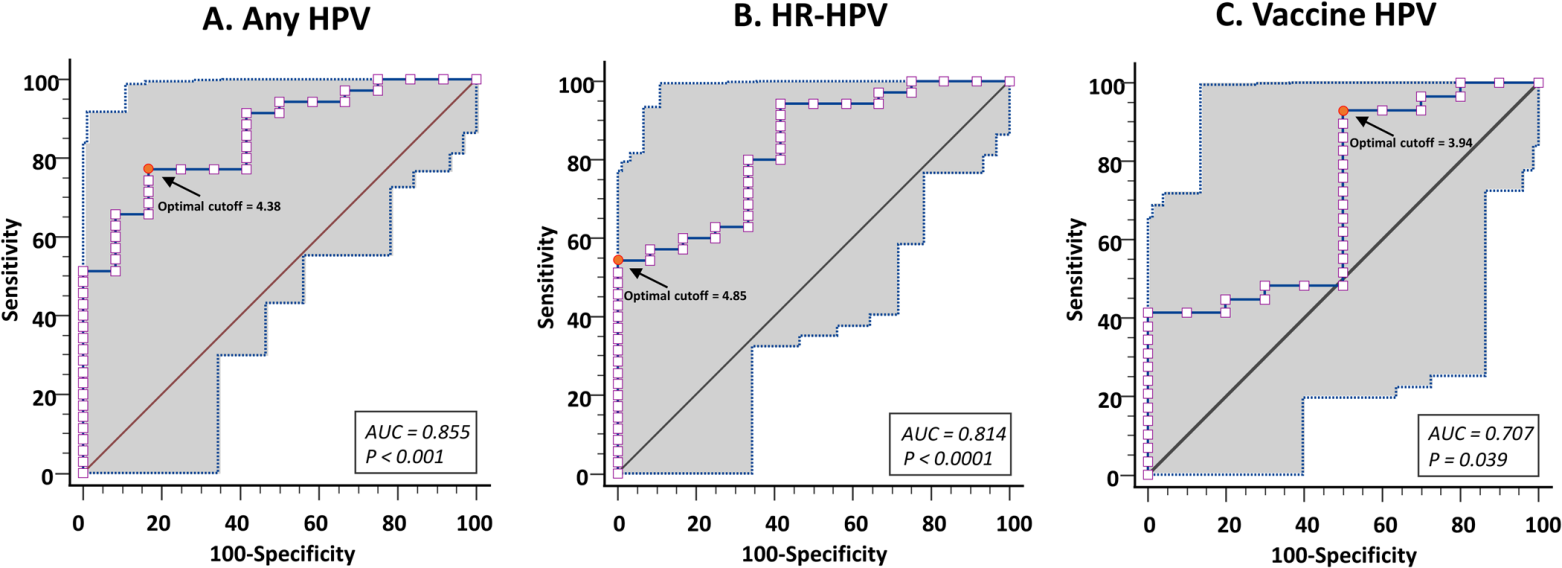
Empirical ROC curves drawn from any HPV (A), HR‐HPV (B) and vaccine HPV (C) viral load data for identifying ICC from high‐grade precancerous and cancerous lesions. The AUC are draw with the 95% confidence bounds (hatched grey area). The most useful cutoff of the optimal viral load level to predict ICC is calculated according to each ROC curve (full circle). The 45° diagonal line serves as the reference line. Any HPV include the following HPV genotypes (HPV‐6, −11, −16, −18, −26, −31, −33, −35, −39, −45, −51, −52, −53, −56, −58, −59, −66, −68, −73, −81, and −82); High risk‐HPV (HR‐HPV) include the following HR‐HPV genotypes (HPV‐16, −18, −31, −33, −35, −39, −45, −51, −52, −56, −58, −59, and − 68); Gardasil‐9® vaccine HPV include the following HPV genotypes (HPV‐6, −11, −16, −18, −31, −33, −45, −52, and −58). AUC: Area under the ROC curve; HR‐HPV: High risk‐HPV; ICC: Invasive cervical cancers; ROC: Receiver operating characteristics.

ROC curves were created from type‐specific HPV viral load data assessed by BMRT HPV Genotyping Real Time PCR assay of any HPV, HR‐HPV, vaccine HPV, HPV‐45 and HV‐16, to determine the most useful cut‐points for the identification of ICC from high‐grade precancerous and cancerous lesions in biopsy specimens (Figure 1). The AUCs for HPV viral load were calculated for ICC endpoints. The sensitivity, specificity, accuracy and overall accuracy of cumulative type‐specific viral loads of any HPV, HR‐HPV and vaccine HPV, for the identification of ICC from CIN2/3 are depicted in the Table 3.

**Table 3:** Sensitivity, specificity, accuracy and overall accuracy of cumulative type‐specific viral loads of any HPV, HR‐HPV and vaccine HPV, for the identification of invasive cervical cancer from high‐grade precancerous and cancerous lesions in biopsy specimens.

	Sensitivity [95% CI]	Specificity [95% CI]	Accuracyµ [95% CI]	Overall accuracy£
**Any HPV** *	77.1 [59.9–89.6]	83.3 [51.6–97.9]	0.60 [0.34–0.75]	0.78
**HR‐HPV** **	54.3 [36.6–71.2]	100.0 [73.5–100.0]	0.54 [0.34–0.65]	0.66
**Vaccine HPV** ***	93.1 [72.2–99.2]	50.0 [18.7–81.3]	0.43 [0.26–0.58]	0.81

* Any HPV include the following 21 HPV genotypes (HPV‐6, −11, −16, −18, −26, −31, −33, −35, −39, −45, −51, −52, −53, −56, −58, −59, −66, −68, −73, −81, and −82).

** HR‐HPV include the following 13 h‐HPV genotypes (HPV‐16, −18, −31, −33, −35, −39, −45, −51, −52, −56, −58, −59, and − 68).

*** Gardasil‐9® vaccine HPV include the following HPV genotypes (HPV‐6, −11, −16, −18, −31, −33, −45, −52, and −58).

^µ^ The accuracy was estimated by Youden's J index (J = Sensitivity + Specificity − 1) [19].

^£^ The overall accuracy taken into account the disease prevalence was calculated using the following formula: (Prevalence) (Sensitivity) + (1‐prévalence) (Specificity) [20]

Abbreviations: CI, Confidence interval; HR‐HPV, High risk‐HPV.

According to the ROC curve analysis, any HPV viral load had an optimal cutoff of 4.38 copies/10,000 cells (log‐transformed) (95% CI = 3.93–4.79) with Youden's J index of 0.60 (95% CI = 0.34–0.75) and an overall accuracy of 0.78. At this cutoff level, the HPV viral load was able to identify ICC with a sensitivity of 77.1% (95% CI = 59.9–89.6), a specificity of 83.3% (95% CI = 51.6–97.9), a PLR of 4.63 (95% CI = 1.29–16.61), a NLR of 0.27 (95% CI = 0.14–0.53), a PPV of 93.1% (95% CI = 79.0–98.0), a NPV of 55.6% (95% CI = 39.3–70.7), and an AUC of 0.855 ± 0.057 (95% CI = 0.721–0.940; *p* < 0.001).

For HR‐HPV viral load, the optimal cutoff was 4.85 copies/10,000 cells (95% CI = 4.68–4.85) with Youden's J index of 0.54 (95% CI = 0.34–0.65) and an overall accuracy of 0.66. At this cutoff level, the HPV viral load was able to identify ICC with a sensitivity of 54.3% (95% CI = 36.6–71.2), a specificity of 100.0% (95% CI = 73.5–100.0), a PLR of 6.51 (95% CI = 0.97–43.60), a NLR of 0.50 (95% CI = 0.33–0.74), a PPV of 95.0% (95% CI = 74.0–99.2), a NPV of 40.7% (95% CI = 31.6–50.6), and an AUC of 0.814 ± 0.069 (95% CI = 0.674–0.913; *p* < 0.001).

For vaccine HPV, the optimal cutoff was 3.94 copies/10,000 cells (95% CI = 3.13–4.85) with Youden's J index of 0.43 (95% CI = 0.26–0.58) and an overall accuracy of 0.81. At this cutoff level, the HPV viral load was able to identify ICC with a sensitivity of 93.1% (95% CI = 72.2–99.2), a specificity of 50.0% (95% CI = 18.7–81.3), a PLR of 1.86 (95% CI = 0.99–3.49), a NLR of 0.14 (95% CI = 0.03–0.60), a PPV of 84.4% (95% CI = 74.2–91.0), a NPV of 71.4% (95% CI = 36.4–91.6), and an AUC of 0.707 ± 0.100 (95% CI = 0.540–0.841; *p *< 0.04).

For the main study HR‐HPV viral loads (supplementary file), HPV‐45 had an AUC of 0.713 ± 0.133 (95% CI = 0.471–0.890; not significant) and an optimal cut‐point of 3.93 copies/10,000 cells (sensitivity = 66.7%, specificity = 80.0%); HPV‐16 had an AUC of 0.589 ± 0.137 (95% CI = 0.338–0.811; *p* = 0.081, not significant) and an optimal cut‐point of 4.79 copies/10,000 cells (sensitivity = 49.9%, specificity = 100.0%); and the sum HPV‐45 plus HPV‐16 had an AUC of 0.688 ± 0.108 (95% CI = 0.497–0.841; not significant) and an optimal cut‐point of 4.79 copies/10,000 cells (sensitivity = 37.5%, specificity = 100.0%).

## 4 Discussion

The present study was conducted to evaluate possible association between the cumulative type‐specific viral loads of the 21 HPV types detected by BMRT HPV Genotyping Real Time PCR assay, and the degree of advanced cervical lesions. In addition, the optimal cutoffs for type‐specific HPV viral loads were defined according to the best diagnosis accuracy, and the ability of cumulative HPV viral loads to discriminate ICC from high‐grade precancerous (CIN2/3) lesions was evaluated by constructing ROC curves according to each cutoff. The first noteworthy result is that the cumulative HPV viral loads of any HPV and HR‐HPV, and to a lesser extent vaccine HPV, were significantly higher in cancerous lesions (ICC), including ADC as well as SCC, than in precancerous lesions (CIN2/3). This finding strongly supports the link between high HPV viral load levels to increased risk of cervical cancer. The second remarkable result is that cumulative viral loads of any HPV and HR‐HPV possessed a good discriminatory ability to differentiate between advanced precancerous lesions (CIN2/3) and ICC, with significantly elevated AUC ranging between 0.80 and 0.90 and useful optimal cutoff levels for the log‐transformed cumulative viral loads of any HPV and HR‐HPV at 4.38 and 4.85 copies per 10,000 cells, respectively. These features indicate that these cutoffs provide viable triage for the risk of ICC in advanced precancerous and cancerous cervical lesions. Taken together, our observations show that cervical cancer may result in an increment of HPV viral load, which is associated with both ADC and SCC malignancies, and that cumulative HPV viral loads assessed by molecular biology in cervical tissue may constitute a relevant biomarker associated with the severity of HPV‐related cervical lesions. Finally, HPV viral load in cervical tissue could be used as a triage tool for aggressive ICC in advanced cervical lesions to determine the group of patients who require mandatory adapted treatment.

In the present series, the cumulative HPV viral loads of any HPV and HR‐HPV, and to a lesser extent vaccine HPV, in study cancerous lesions (ICC) were significantly higher than those in study precancerous lesions (CIN2/3), strongly suggesting positive association between the levels of tissue HPV viral loads and the risk of cervical cancer, thus the tumour aggressiveness. These observations are, first of all, reminiscent to the fact that high genital HPV viral load is correlated with persistent infection leading to increased cervical cancer risk, and constitutes, with ad hoc threshold, a discriminative marker for the development of precancerous and cancerous lesions of the cervix [21, 22, 23]. Furthermore, numerous studies agree that there is an overall genital HPV viral load increases with the lesion severity [11, 24, 25, 26] and worse clinical outcome [8], suggesting that HPV viral load could be as a diagnostic marker of prevalent cervical lesion [27, 28, 29].

Studies on cervical HPV viral load and HPV‐related cancer in sub‐Saharan Africa are limited, and remain relatively difficult to compare, due to considerable methodological heterogeneity and the variety of populations included. According to reports on cytology samples taken for screening/referral purposes, higher genital HPV viral load was linked to squamous cell inflammation in Gabon [30], multiple cervical HPV infections in South Africa [31] and Kenya [32], and higher CIN grade or cervical cancer stage in Burkina Faso [33, 34], South Africa [31], Senegal [35] and Kenya [36], especially in HIV‐infected women [32, 33, 34, 35, 36, 37]. Genital viral load of the α−9 HPV‐16 was the main primary driver of the observed increase in high‐grade cervical dysplasia or ICC in Burkina Faso [33] and Senegal [35], but inconsistently in other African settings [32, 37, 38]. Furthermore, total genital viral load of the α−7 HPV‐18 was higher in women with squamous intraepithelial lesions than in women with normal cytology in Burkina Faso [34], although the association between HPV‐18 viral load and cervical lesion severity is controversial [39, 40]. Only few studies for tissue HPV viral load by quantitative PCR involved cervical biopsy specimens collected from patients living in South Africa [40, 41] and Burkina Faso [40] for diagnostic purposes to determine the cancer stage and type to decide what treatment plan they should receive. In South Africa, HPV viral load in cervical biopsy specimen was higher in case of multiple HPV infections [41]. HPV‐16 was the most frequently detected type associated with ICC and demonstrated the highest tissue viral load [41]. In South Africa and Burkina Faso, a high baseline HPV‐16 viral load in cervical biopsies was significantly associated with persistence of, or progression to ≥CIN2 at endline, while these findings could not be observed for HPV‐18, demonstrating that HPV‐16 viral load is a powerful marker of ≥CIN2 in HIV‐infected African women [40].

The diagnostic capacity and accuracy of intra‐tissue HPV viral load, as a potential triage biomarker, was further evaluated to distinguish CIN2/3 from ICC in study included biopsy specimens, using ROC curves analysis. Indeed, in recent years, there has been overwhelming evidence that primary HPV molecular testing has become the cornerstone of cervical cancer secondary prevention, as the optimal, most efficient screening approach for cervical cancer with outstanding sensitivity in both low‐ and high‐ resource settings [42, 43, 44]. The availability of new robust HPV molecular testing platforms enables primary HPV screening to be implemented in most African countries [2, 4, 43, 44]. HPV molecular testing has the highest sensitivity of any known approach to cervical precancer and cancer screening [43, 45]. Furthermore, HPV testing has the highest negative predictive value for cervical precancerous lesions, providing greater and lengthier reassurance against future risk of developing pre‐cancer/cancer [46]. The main disadvantage of HPV testing is its moderate specificity arising from its inability to fully discriminate between transient and persistent infections with one of the 13 h‐HPV genotypes [42, 47]. Thus, only a subset of women infected with HR‐HPV progress to develop invasive cervical cancer since most infections will spontaneously clear [48]. This challenge is particularly marked in women living with HIV who have high rates of HPV infections, a situation particularly frequent in sub‐Saharan Africa [4, 49]. The recently 2021‐revised WHO guidelines for screening and treatment of cervical precancer lesions argue for the need for triage tests after primary HPV molecular testing [4]. Only a limited number of routine biomarkers may be used in Africa [43] among numerous potential triage biomarkers [6, 47, 50]. In practice, the choice of triage test essentially depends on the availability of health care and laboratory facilities [43]. Furthermore, multiple compounding factors in low‐ and middle‐ income countries, such as intrinsic weaknesses of cervical cancer prevention programs with limited resources, particularly regarding good‑quality cytological testing and colposcopy evaluation [12], may contribute to limiting the access of convenient triage markers even in case of correct HPV molecular testing, thus accentuating the high burden of cervical cancer in sub‐Sahara Africa [51]. In the particular context of sub‐Saharan Africa, we herein evaluated possible diagnosis capabilities of HPV viral load in biopsy tissue lesions, as assessed by BMRT HPV Genotyping Real Time PCR assay. Results obtained in this study indicate that cumulative HPV viral loads in biopsy tissue specimens of precancerous or cancerous lesions of any HPV and HR‐HPV, and to a lesser extent vaccine HPV, could be reliable markers of ICC when considering advanced stages of histological cervical lesions. Lower association between histological grades and vaccine HPV could be due to the frequent detection of non‐vaccine‐covered HPV in study women. Finally, the cumulative viral loads of any HPV covered by the Bioperfectus BMRT HPV assay could constitute a reliable marker of ICC in advanced precancerous and cancerous cervical lesions. The threshold of 4.38 log HPV DNA copies/10,000 cells to predict prevalent or incident ICC is in line with the previous reported tissue viral loads for ≥CIN2 in Senegal [40] and South Africa [41].

Our study has several limitations that need to be considered. First, the representativeness of the included study population is not ensured, even for the country Mauritania. Such inclusion bias could be extended in other urban and rural remote areas in Mauritania, where no women were included. Second, the small sample size of our study population may have introduced a selection bias because included precancer and cancer cases could represent a select group of women who were healthy enough to participate in study enrolment, and therefore likely had less severe cancer stages. Third, the lack of ≤CIN1 in our study biopsies clearly makes it impossible to assess the value of the HPV viral load biomarker throughout the natural history of the HR‐HPV‐related disease, with the risk of overestimating the value of this biomarker only at advanced or very advanced stages. Fourth, we hypothesized that the ratio of cases in the positive (ICC) and negative (CIN2/3) groups reflects the prevalence of the disease, which can lead to bias for the calculation of PPV and NPV. Finally, the preselection of pathologic cervical biopsy material decreases the probability of finding HPV genotypes not associated with high‐grade lesions or cancer.

## 5 Conclusion

HPV molecular testing is the optimal, most efficient screening approach for both low‐ and high‐ resource settings and has the highest sensitivity and negative predictive values of any known approach to cervical cancer screening [6, 7]. Otherwise, cumulative HPV viral load in cervical tissue may also constitute a relevant biomarker associated with the severity of HPV‐related cervical lesions, and could be used as a triage tool for aggressive ICC in advanced cervical lesions.

Integrating HPV viral load into routine screening promises to optimize resource allocation and diminish the burden of prevention programs by reducing costs, time, and the need for invasive biopsies. In HPV‐based screening, high‐risk HPV‐positive individuals with elevated viral loads (especially HPV‐16 and HPV‐18) would warrant immediate colposcopy. Conversely, those with lower viral loads could be managed with extended‐interval HPV retesting, thereby minimizing unnecessary colposcopies for transient infections and yielding substantial cost savings. This risk‐stratified approach streamlines clinical workflow, shortens waiting times for high‐risk patients, and optimizes colposcopy resource utilization. Furthermore, viral load monitoring posttreatment for high‐grade cervical lesions (CIN2/3) can detect persistent disease or recurrence, guiding targeted follow‐up and enhancing cost‐effectiveness.

While initial assay costs must be considered, particularly in resource‐limited settings, the long‐term benefits of reduced unnecessary procedures are significant. Successful implementation necessitates robust integration with existing laboratory infrastructure, investment in appropriate technology, and stringent quality control protocols to ensure result accuracy and reliability.

## Author Contributions

**Mariem Salma Abdoudaim:** Conceptualization; data curation; formal analysis; investigation; methodology; software; writing – original draft; writing – review and editing. **Laurent Bélec:** Formal analysis; methodology; software; supervision; validation; writing – original draft; writing – review and editing. **Mohamed Lemine Cheikh Brahim Ahmed:** Conceptualization; data curation; investigation; validation; visualization; writing – review and editing. **Nacer Dine Mohamed Baba:** Conceptualization; data curation; investigation; visualization; writing – review and editing. **Ralph‐Sydney Mboumba Bouassa:** Formal analysis; methodology; software; writing – original draft; writing – review and editing. **Mohamed Vall Mohamed Abdellahi:** Conceptualization; project administration; supervision; validation; writing – review and editing.

## Ethics Statement

This study was conducted in accordance with the ethical guidelines of the World Medical Association Declaration of Helsinki. Signed informed written consent was obtained by each participant after explaining the purpose of the cervical biopsy for histological and virological analyses. This study study is with a direct individual benefit, with the histological diagnosis of suspected cervical lesions and virological diagnosis of HPV detection and genotyping. The protocol was reviewed and approved by the Institutional Review Board and Ethics Committee of the Nouakchott University, Nouakchott, Mauritania (approval no. 004/CE/UN on 27 January 2022).

## Consent

Informed written consent was obtained from all participants to be involved in the study.

## Conflicts of Interest

The authors declare no conflict of interest.

## Transparency Statement

The lead author Mariem Salma Abdoudaim affirms that this manuscript is an honest, accurate, and transparent account of the study being reported; that no important aspects of the study have been omitted; and that any discrepancies from the study as planned (and, if relevant, registered) have been explained.

## Supporting information

Supplementary file.

## Data Availability

The data that support the findings of this study are available from the corresponding author upon reasonable request.
