# Supplementary material for: Human Papillomavirus Viral Load as Triage Biomarker for High‐Grade Cervical Lesions and Invasive Cervical Carcinoma: A Cross‐Sectional Study
Source: Health Sci Rep. 2025 Nov 17;8(11):e71524. doi: 10.1002/hsr2.71524 (PMC12620665; doi:10.1002/hsr2.71524)
Supplement: Supplementary file 1 — Supplementary file. [file HSR2-8-e71524-s001.pdf]

ROC curve

Variable	Viral_load_of_HPV_45<br>Viral_load_of_HPV-45
Classification variable	Disease<br>Disease

Sample size	20
Positive group [a]	15 (75,00%)
Negative group [b]	5 (25,00%)

[a] Disease = 1
[b] Disease = 0

Disease prevalence (%)	75,0

Area under the ROC curve (AUC)

Area under the ROC curve (AUC)	0,713
Standard Error [a]	0,133
95% Confidence interval [b]	0,471 to 0,890
95% Bootstrap CI [c]	0,452 to 0,920
z statistic	1,607
Significance level P (Area=0.5)	0,1081

[a] DeLong et al., 1988
[b] Binomial exact
[c] BC$_a$ bootstrap confidence interval (1000 iterations; random number seed: 978).

Youden index

Youden index J	0,4667
95% Confidence interval [a]	0,2000 to 0,7333
Associated criterion	>3,936262342
95% Confidence interval [a]	>3,102090526 to >4,396931275
Sensitivity	66,67
Specificity	80,00

[a] BC$_a$ bootstrap confidence interval (1000 iterations; random number seed: 978).

Summary Table

Estimated specificity at fixed sensitivity			
Sensitivity	Specificity	95% CI [a]	Criterion
80,00	40,00	0,00 to 100,00	>3,651278014
90,00	40,00	0,00 to 100,00	>3,235467517
95,00	0,00	20,00 to 20,00	>1,633468456
97,50	0,00	20,00 to 20,00	>1,633468456
99,00	0,00	20,00 to 20,00	>1,633468456
Estimated sensitivity at fixed specificity			
Specificity	Sensitivity	95% CI [a]	Criterion
80,00	66,67	30,00 to 100,00	>3,936262342
90,00	36,67	6,67 to 73,33	>4,354681265
95,00	35,00	6,67 to 66,67	>4,37580627
97,50	34,17	0,00 to 0,00	>4,386368773
99,00	33,67	0,00 to 0,00	>4,392706274

[a] BC$_a$ bootstrap confidence interval (1000 iterations; random number seed: 978).

Criterion values and coordinates of the ROC curve [Hide]

Criterion	Sensitivity	95% CI	Specificity	95% CI	+LR	95% CI	-LR	95% CI	+PV	95% CI	-PV	95% CI
≥1,633468456	100,00	78,2 - 100,0	0,00	0,0 - 52,2	1,00	1,00 - 1,00			75,0	75,0 - 75,0		
>1,633468456	93,33	68,1 - 99,8	0,00	0,0 - 52,2	0,93	0,82 - 1,07			73,7	71,0 - 76,2	0,0	
>3,050766311	93,33	68,1 - 99,8	20,00	0,5 - 71,6	1,17	0,74 - 1,85	0,33	0,025 - 4,40	77,8	68,9 - 84,7	50,0	7,0 - 93,0
>3,102090526	93,33	68,1 - 99,8	40,00	5,3 - 85,3	1,56	0,75 - 3,22	0,17	0,019 - 1,47	82,4	69,3 - 90,6	66,7	18,5 - 94,6
>3,368844507	86,67	59,5 - 98,3	40,00	5,3 - 85,3	1,44	0,69 - 3,04	0,33	0,062 - 1,79	81,2	67,3 - 90,1	50,0	15,7 - 84,3
>3,651278014	80,00	51,9 - 95,7	40,00	5,3 - 85,3	1,33	0,62 - 2,85	0,50	0,11 - 2,19	80,0	65,2 - 89,5	40,0	13,2 - 74,5
>3,670153045	73,33	44,9 - 92,2	40,00	5,3 - 85,3	1,22	0,56 - 2,66	0,67	0,17 - 2,60	78,6	62,7 - 88,9	33,3	11,3 - 66,1
>3,738463439	66,67	38,4 - 88,2	40,00	5,3 - 85,3	1,11	0,50 - 2,47	0,83	0,23 - 3,03	76,9	60,0 - 88,1	28,6	9,9 - 59,2
>3,923917223	66,67	38,4 - 88,2	60,00	14,7 - 94,7	1,67	0,54 - 5,17	0,56	0,20 - 1,53	83,3	61,7 - 93,9	37,5	17,9 - 62,3
>3,936262342	66,67	38,4 - 88,2	80,00	28,4 - 99,5	3,33	0,56 - 19,95	0,42	0,18 - 0,96	90,9	62,6 - 98,4	44,4	25,7 - 64,9
>4,03702788	60,00	32,3 - 83,7	80,00	28,4 - 99,5	3,00	0,50 - 18,17	0,50	0,23 - 1,07	90,0	59,8 - 98,2	40,0	23,8 - 58,7
>4,049605613	53,33	26,6 - 78,7	80,00	28,4 - 99,5	2,67	0,43 - 16,39	0,58	0,29 - 1,17	88,9	56,6 - 98,0	36,4	22,2 - 53,4
>4,271097792	46,67	21,3 - 73,4	80,00	28,4 - 99,5	2,33	0,37 - 14,61	0,67	0,35 - 1,27	87,5	52,8 - 97,8	33,3	20,8 - 48,8
>4,312431256	40,00	16,3 - 67,7	80,00	28,4 - 99,5	2,00	0,31 - 12,84	0,75	0,41 - 1,37	85,7	48,3 - 97,5	30,8	19,6 - 44,8
>4,396931275	33,33	11,8 - 61,6	100,00	47,8 - 100,0			0,67	0,47 - 0,95	100,0		33,3	25,9 - 41,7
>4,580822374	26,67	7,8 - 55,1	100,00	47,8 - 100,0			0,73	0,54 - 1,00	100,0		31,2	25,1 - 38,1
>4,614580867	20,00	4,3 - 48,1	100,00	47,8 - 100,0			0,80	0,62 - 1,03	100,0		29,4	24,4 - 34,9
>5,92243012	13,33	1,7 - 40,5	100,00	47,8 - 100,0			0,87	0,71 - 1,06	100,0		27,8	24,0 - 31,9
>6,277175161	6,67	0,2 - 31,9	100,00	47,8 - 100,0			0,93	0,82 - 1,07	100,0		26,3	23,8 - 29,0
>8,123496236	0,00	0,0 - 21,8	100,00	47,8 - 100,0			1,00	1,00 - 1,00			25,0	25,0 - 25,0

ROC curve

Variable	Viral_load_of_HPV_16
Classification variable	Disease<br>Disease

Sample size	18
Positive group [a]	14 (77,78%)
Negative group [b]	4 (22,22%)

[a] Disease = 1
[b] Disease = 0

Disease prevalence (%)	77,8

Area under the ROC curve (AUC)

Area under the ROC curve (AUC)	0,589
Standard Error [a]	0,137
95% Confidence interval [b]	0,338 to 0,811
95% Bootstrap CI [c]	0,321 to 0,823
z statistic	0,650
Significance level P (Area=0.5)	0,5159

[a] DeLong et al., 1988
[b] Binomial exact
[c] BC_a bootstrap confidence interval (1000 iterations; random number seed: 978).

Youden index

Youden index J	0,4286
95% Confidence interval [a]	0,2500 to 0,5714
Associated criterion	>4,795330722
95% Confidence interval [a]	>4,62930764 to >4,795330722
Sensitivity	42,86
Specificity	100,00

[a] BC_a bootstrap confidence interval (1000 iterations; random number seed: 978).

Summary Table

Estimated specificity at fixed sensitivity			
Sensitivity	Specificity	95% CI [a]	Criterion
80,00	0,00	0,00 to 25,00	>3,602324384
90,00	0,00	0,00 to 50,00	>3,059458701
95,00	0,00	0,00 to 25,00	>2,890979597
97,50	0,00	0,00 to 25,00	>2,890979597
99,00	0,00	0,00 to 0,00	>2,890979597
Estimated sensitivity at fixed specificity			
Specificity	Sensitivity	95% CI [a]	Criterion
80,00	42,86	11,48 to 64,29	>4,748826622
90,00	42,86	11,48 to 64,29	>4,772078672
95,00	42,86	11,48 to 64,29	>4,783704697
97,50	42,86	0,00 to 0,00	>4,78951771
99,00	42,86	0,00 to 0,00	>4,793005517

[a] BC_a bootstrap confidence interval (1000 iterations; random number seed: 978).

Criterion values and coordinates of the ROC curve [Hide]

Criterion	Sensitivity	95% CI	Specificity	95% CI	+LR	95% CI	-LR	95% CI	+PV	95% CI	-PV	95% CI
≥2,890979597	100,00	76,8 - 100,0	0,00	0,0 - 60,2	1,00	1,00 - 1,00			77,8	77,8 - 77,8		
>2,890979597	92,86	66,1 - 99,8	0,00	0,0 - 60,2	0,93	0,80 - 1,07			76,5	73,8 - 79,0	0,0	
>3,312177356	85,71	57,2 - 98,2	0,00	0,0 - 60,2	0,86	0,69 - 1,06			75,0	70,8 - 78,8	0,0	
>3,674861141	78,57	49,2 - 95,3	0,00	0,0 - 60,2	0,79	0,60 - 1,03			73,3	67,7 - 78,3	0,0	
>3,872971631	78,57	49,2 - 95,3	25,00	0,6 - 80,6	1,05	0,56 - 1,96	0,86	0,12 - 6,16	78,6	66,2 - 87,3	25,0	4,4 - 70,5
>3,890979597	71,43	41,9 - 91,6	25,00	0,6 - 80,6	0,95	0,49 - 1,83	1,14	0,17 - 7,56	76,9	63,4 - 86,5	20,0	3,6 - 62,3
>4,268624768	64,29	35,1 - 87,2	25,00	0,6 - 80,6	0,86	0,43 - 1,70	1,43	0,23 - 8,97	75,0	60,1 - 85,6	16,7	3,1 - 55,7
>4,545962394	57,14	28,9 - 82,3	25,00	0,6 - 80,6	0,76	0,37 - 1,57	1,71	0,28 - 10,39	72,7	56,4 - 84,6	14,3	2,7 - 50,3
>4,606058749	57,14	28,9 - 82,3	50,00	6,8 - 93,2	1,14	0,39 - 3,37	0,86	0,27 - 2,71	80,0	57,6 - 92,2	25,0	9,5 - 51,3
>4,62930764	57,14	28,9 - 82,3	75,00	19,4 - 99,4	2,29	0,39 - 13,25	0,57	0,25 - 1,31	88,9	58,0 - 97,9	33,3	17,9 - 53,4
>4,680063427	50,00	23,0 - 77,0	75,00	19,4 - 99,4	2,00	0,34 - 11,82	0,67	0,31 - 1,44	87,5	54,2 - 97,6	30,0	16,5 - 48,1
>4,737200597	42,86	17,7 - 71,1	75,00	19,4 - 99,4	1,71	0,28 - 10,39	0,76	0,37 - 1,57	85,7	49,7 - 97,3	27,3	15,4 - 43,6
>4,795330722	42,86	17,7 - 71,1	100,00	39,8 - 100,0			0,57	0,36 - 0,90	100,0		33,3	24,1 - 44,0
>4,884330811	35,71	12,8 - 64,9	100,00	39,8 - 100,0			0,64	0,44 - 0,95	100,0		30,8	23,1 - 39,6
>4,892901196	28,57	8,4 - 58,1	100,00	39,8 - 100,0			0,71	0,51 - 0,99	100,0		28,6	22,3 - 35,8
>5,151896691	21,43	4,7 - 50,8	100,00	39,8 - 100,0			0,79	0,60 - 1,03	100,0		26,7	21,7 - 32,3
>5,570855706	14,29	1,8 - 42,8	100,00	39,8 - 100,0			0,86	0,69 - 1,06	100,0		25,0	21,2 - 29,2
>6,45786056	7,14	0,2 - 33,9	100,00	39,8 - 100,0			0,93	0,80 - 1,07	100,0		23,5	21,0 - 26,2
>7,895891679	0,00	0,0 - 23,2	100,00	39,8 - 100,0			1,00	1,00 - 1,00			22,2	22,2 - 22,2

ROC curve

Variable	Viral_load_of_HPV_45_16<br>Viral_load_of_HPV_45+16
Classification variable	Disease<br>Disease

Sample size	31
Positive group [a]	24 (77,42%)
Negative group [b]	7 (22,58%)

[a] Disease = 1
[b] Disease = 0

Disease prevalence (%)	77,4

Area under the ROC curve (AUC)

Area under the ROC curve (AUC)	0,688
Standard Error [a]	0,108
95% Confidence interval [b]	0,497 to 0,841
95% Bootstrap CI [c]	0,453 to 0,867
z statistic	1,744
Significance level P (Area=0.5)	0,0812

[a] DeLong et al., 1988
[b] Binomial exact
[c] BC$_a$ bootstrap confidence interval (1000 iterations; random number seed: 978).

Youden index

Youden index J	0,3750
95% Confidence interval [a]	0,1786 to 0,5060
Associated criterion	>4,795330722
95% Confidence interval [a]	>4,396931275 to >4,795330722
Sensitivity	37,50
Specificity	100,00

[a] BC$_a$ bootstrap confidence interval (1000 iterations; random number seed: 978).

Summary Table

Estimated specificity at fixed sensitivity			
Sensitivity	Specificity	95% CI [a]	Criterion
80,00	28,57	0,00 to 85,71	>3,887378004
90,00	14,29	0,00 to 71,43	>3,491251161
95,00	14,29	0,00 to 57,14	>3,323510786
97,50	14,29	14,29 to 28,57	>3,228142624
99,00	14,29	14,29 to 28,57	>3,152511365
**Estimated sensitivity at fixed specificity**			
Specificity	Sensitivity	95% CI [a]	Criterion
80,00	45,83	16,67 to 79,17	>4,609913534
90,00	37,50	16,67 to 58,43	>4,778612703
95,00	37,50	16,67 to 58,33	>4,786971713
97,50	37,50	0,00 to 0,00	>4,791151217
99,00	37,50	0,00 to 0,00	>4,79365892

[a] BC$_a$ bootstrap confidence interval (1000 iterations; random number seed: 978).

Criterion values and coordinates of the ROC curve [Hide]

Criterion	Sensitivity	95% CI	Specificity	95% CI	+LR	95% CI	-LR	95% CI	+PV	95% CI	-PV	95% CI
≥3,102090526	100,00	85,8 - 100,0	0,00	0,0 - 41,0	1,00	1,00 - 1,00			77,4	77,4 - 77,4		
>3,102090526	100,00	85,8 - 100,0	14,29	0,4 - 57,9	1,17	0,86 - 1,58	0,00		80,0	74,7 - 84,4	100,0	
>3,312177356	95,83	78,9 - 99,9	14,29	0,4 - 57,9	1,12	0,82 - 1,53	0,29	0,021 - 4,09	79,3	73,7 - 84,0	50,0	6,7 - 93,3
>3,368844507	91,67	73,0 - 99,0	14,29	0,4 - 57,9	1,07	0,77 - 1,48	0,58	0,062 - 5,52	78,6	72,6 - 83,5	33,3	5,0 - 82,6
>3,674861141	87,50	67,6 - 97,3	14,29	0,4 - 57,9	1,02	0,73 - 1,43	0,88	0,11 - 7,15	77,8	71,4 - 83,1	25,0	3,9 - 73,1
>3,738463439	83,33	62,6 - 95,3	14,29	0,4 - 57,9	0,97	0,68 - 1,38	1,17	0,15 - 8,82	76,9	70,1 - 82,6	20,0	3,2 - 65,4
>3,872971631	83,33	62,6 - 95,3	28,57	3,7 - 71,0	1,17	0,71 - 1,93	0,58	0,13 - 2,55	80,0	70,8 - 86,9	33,3	10,3 - 68,6
>3,890979597	79,17	57,8 - 92,9	28,57	3,7 - 71,0	1,11	0,66 - 1,85	0,73	0,18 - 2,98	79,2	69,5 - 86,4	28,6	8,9 - 62,0
>3,923917223	79,17	57,8 - 92,9	42,86	9,9 - 81,6	1,39	0,71 - 2,72	0,49	0,15 - 1,55	82,6	70,8 - 90,3	37,5	15,9 - 65,6
>3,936262342	79,17	57,8 - 92,9	57,14	18,4 - 90,1	1,85	0,77 - 4,45	0,36	0,13 - 1,00	86,4	72,4 - 93,9	44,4	22,6 - 68,7
>4,03702788	75,00	53,3 - 90,2	57,14	18,4 - 90,1	1,75	0,72 - 4,24	0,44	0,17 - 1,12	85,7	71,2 - 93,6	40,0	20,6 - 63,2
>4,049605613	70,83	48,9 - 87,4	57,14	18,4 - 90,1	1,65	0,68 - 4,04	0,51	0,21 - 1,25	85,0	69,9 - 93,3	36,4	18,9 - 58,3
>4,268624768	66,67	44,7 - 84,4	57,14	18,4 - 90,1	1,56	0,63 - 3,83	0,58	0,25 - 1,37	84,2	68,4 - 92,9	33,3	17,5 - 54,0
>4,271097792	62,50	40,6 - 81,2	57,14	18,4 - 90,1	1,46	0,59 - 3,62	0,66	0,29 - 1,50	83,3	66,8 - 92,5	30,8	16,3 - 50,3
>4,312431256	58,33	36,6 - 77,9	57,14	18,4 - 90,1	1,36	0,54 - 3,41	0,73	0,33 - 1,62	82,4	65,0 - 92,1	28,6	15,3 - 47,0
>4,396931275	54,17	32,8 - 74,4	71,43	29,0 - 96,3	1,90	0,56 - 6,47	0,64	0,34 - 1,22	86,7	65,6 - 95,7	31,3	19,3 - 46,3
>4,545962394	50,00	29,1 - 70,9	71,43	29,0 - 96,3	1,75	0,51 - 6,03	0,70	0,38 - 1,30	85,7	63,5 - 95,4	29,4	18,4 - 43,6
>4,580822374	45,83	25,6 - 67,2	71,43	29,0 - 96,3	1,60	0,46 - 5,60	0,76	0,42 - 1,38	84,6	61,2 - 95,0	27,8	17,5 - 41,1
>4,62930764	45,83	25,6 - 67,2	85,71	42,1 - 99,6	3,21	0,50 - 20,73	0,63	0,39 - 1,02	91,7	63,0 - 98,6	31,6	22,3 - 42,6
>4,680063427	41,67	22,1 - 63,4	85,71	42,1 - 99,6	2,92	0,45 - 19,03	0,68	0,43 - 1,07	90,9	60,5 - 98,5	30,0	21,4 - 40,3
>4,771447838	37,50	18,8 - 59,4	85,71	42,1 - 99,6	2,62	0,40 - 17,32	0,73	0,47 - 1,12	90,0	57,7 - 98,3	28,6	20,6 - 38,1
>4,795330722	37,50	18,8 - 59,4	100,00	59,0 - 100,0			0,63	0,46 - 0,85	100,0		31,8	25,5 - 38,9

>4,884330811	33,33	15,6 - 55,3	100,00	59,0 - 100,0			0,67	0,50 - 0,88	100,0		30,4	24,8 - 36,7
>4,892901196	29,17	12,6 - 51,1	100,00	59,0 - 100,0			0,71	0,55 - 0,92	100,0		29,2	24,2 - 34,7
>5,151896691	25,00	9,8 - 46,7	100,00	59,0 - 100,0			0,75	0,60 - 0,94	100,0		28,0	23,6 - 32,9
>5,570905868	20,83	7,1 - 42,2	100,00	59,0 - 100,0			0,79	0,64 - 0,97	100,0		26,9	23,1 - 31,1
>5,922833888	16,67	4,7 - 37,4	100,00	59,0 - 100,0			0,83	0,70 - 1,00	100,0		25,9	22,6 - 29,5
>6,277175161	12,50	2,7 - 32,4	100,00	59,0 - 100,0			0,88	0,75 - 1,02	100,0		25,0	22,3 - 27,9
>6,458568054	8,33	1,0 - 27,0	100,00	59,0 - 100,0			0,92	0,81 - 1,03	100,0		24,1	22,0 - 26,4
>7,896118853	4,17	0,1 - 21,1	100,00	59,0 - 100,0			0,96	0,88 - 1,04	100,0		23,3	21,9 - 24,9
>8,123496236	0,00	0,0 - 14,2	100,00	59,0 - 100,0			1,00	1,00 - 1,00			22,6	22,6 - 22,6
